# The long-term outcomes of alternating chemoradiotherapy for locoregionally advanced nasopharyngeal carcinoma: a multiinstitutional phase II study

**DOI:** 10.1002/cam4.469

**Published:** 2015-05-20

**Authors:** Nobukazu Fuwa, Takeshi Kodaira, Takashi Daimon, Tomokazu Yoshizaki

**Affiliations:** 1 Department of Radiology, Hyogo Ion Beam Medical CenterHyogo, Japan; 2 Department of Radiation Oncology, Aichi Cancer CenterAichi, Japan; 3 Department of Biostatistics, Hyogo College of MedicineHyogo, Japan; 4 Department of Otorhinolaryngology, Kanazawa UniversityIshikawa, Japan

**Keywords:** 5-fluorouracil, alternating chemoradiotherapy, cisplatin, nasopharyngeal carcinoma, phase 2 study

## Abstract

To examine the long-term outcomes of alternating chemoradiotherapy (ALCRT) for patients with locoregionally advanced nasopharyngeal carcinoma (NPC) and to assess the efficacy of ALCRT for NPC. Patients with stage IIB to IVB, ECOG PS 0–2, 18–70 years-old, and sufficient organ function were eligible for this study. First, chemotherapy, consisting of 5-fluorouracil (800 mg/m^2^ per 24 h on days 1–5) and cisplatin (100 mg/m^2^ per 24 h on day 6), was administered, then a wide field of radiotherapy (36 Gy/20 fraction), chemotherapy, a shrinking field of radiotherapy (34 Gy/17 fraction), and chemotherapy were performed alternately. Between December 2003 and March 2006, 90 patients in 25 facilities were enrolled in this study, 87 patients were finally evaluated. A total of 67 patients (76.1%) completed the course of treatment. The overall survival and the progression-free survival rates at 5 years were 78.04% (95% CI: 69.1∼87.0%), and 68.74% (95% CI: 58.8∼78.7%), respectively. The long-term outcomes of ALCRT for NPC were thought to be promising. ALCRT will be considered to be a controlled trial to compare therapeutic results with those of concurrent chemoradiotherapy for NPC.

## Introduction

Nasopharyngeal carcinoma (NPC) is endemic in Southern China and Southeast Asia 1,2. However, it is a rare disease in most countries including Japan 3. Because of anatomical features, NPC is not suitable for surgery. In addition, the majority of NPC which has wild-type p53 is highly radiosensitive 4. Thus, radiotherapy (RT) plays a central role in the locoregional control of NPC 5,6. Another characteristic of NPC is that distant metastasis is more common than it is in other carcinomas of the head and neck. Therefore, chemotherapy plays an important role in improving treatment outcomes.

In 1998, the American Intergroup 0099 study (IGS 0099), which used three courses each of concurrent chemotherapy (cisplatin [CDDP], 100 mg/m^2^) and adjuvant chemotherapy (AC) (CDDP, 80 mg/m^2^ and 5-fluorouracil [5-FU], 4000 mg/m^2^ per 4 days), was the first study to demonstrate significant improvements in the overall survival (OS) compared with RT alone 7. However, issues have arisen with this treatment method. A higher incidence of adverse events of Grade 3 or higher has been observed in the chemoradiation group than in the radiation alone group (59% vs. 34%). Approximately, about 55% underwent the full course of AC. As a result, chemotherapy reduced distant metastasis. However, it might not be sufficient.

In the Aichi Cancer Center, we conducted alternating chemoradiotherapy (ALCRT) with three courses of FP (5-FU and CDDP) in patients with locally advanced NPC from 1987 and reported promising results with a better compliance 8,9. Considering these results, a phase II study in which 25 facilities participated was initiated in December 2003.

The present study was to evaluate the long-term outcomes and to assess the usefulness of ALCRT in patients with NPC.

## Materials and Methods

Eligible patient for this study was as follows, histologically-proven WHO type I to III NPC, clinical stage IIB-IVB (2003 TNM), age 18–70 year old with a performance score of 0–2 (Eastern Cooperative Oncology Group), WBC count ≥ 3000/mm^3^, neutrophil ≥ 1500/mm^3^, platelet count ≥ 100,000 mm^3^, hemoglobin ≥ 10 g/dL, total bililubin ≤ 2.0 mg/dL, creatinine clearance ≥ 60 mL/min, without physical and mental diseases that were obstacle to receive the protocol therapy, and approved by the Ethics Committee of each facility.

The patients with active multiple cancer, history of radiotherapy and/or surgery for head and neck cancer, obvious infectious diseases, and history of severe drug allergy, were excluded from registration. Extend of disease evaluation included a fiberscopic examination of upper-airway from nose to hypopharynx, chest X-P, liver ultrasonography or CT, bone scan, computed tomography (CT) scan and magnetic resonance image (MRI) scan of the nasopharynx, skullbase, and neck. All patients were required to provide written informed consent before registration.

### Alternating chemoradiotherapy

**Figure 1:**
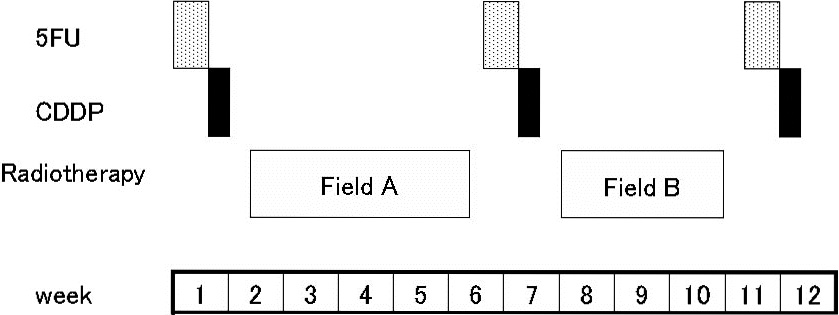
Study design of alternating chemoradiotherapy. 5FU: 5-fluorouracil; 800 mg/m^2^ per day i.v. continuous infusion × 5 days (day 1–5); CDDP: cisplatin, 100 mg/m^2^ per day i.v. continuous infusion (day 6); Field A: large field (from the skull base to the supraclavicular fossa), 1.8 Gy/f, 5 f/w, total tumor dose; 36 Gy; Field B: shrinking field, 2 Gy/f, 5 f/w, total tumor dose; 34 Gy. The interval between Field A and Field B is 10–12 days.

The treatment scheme is shown in Figure1. 5FU at a dose of 800 mg/m^2^ per 24 h was administered intravenously for a 120-h infusion, followed by a 24 h infusion of CDDP at a dose of 100 mg/m^2^ per 24 h (day 6). During the course of ALCRT, chemotherapy was performed before RT, and RT (Field A) was then performed for 4 weeks beginning 2–3 days after the completion of chemotherapy. The second course of chemotherapy was performed 2–3 days after the completion of RT. The second course of RT (Field B) was then performed with a reduced irradiation field 2–3 days after the second chemotherapy. Furthermore, the third course of chemotherapy was performed 2–3 days after the completion of the second course of RT.

Chemotherapy was not performed when serum creatinine levels exceeded 1.5 mg/dL at the scheduled date of chemotherapy. In addition, chemotherapy was postponed for the patients with WBC counts <3000/mm^2^ or platelet counts < 75,000/mm^2^ at the scheduled date of chemotherapy. Moreover, when the patients did not meet the above hematologic criteria (WBC counts ≥ 3000/mm^2^ and platelet counts ≥ 75,000/mm^2^) more than 6 weeks after the last day of previous chemotherapy, the administration of chemotherapy was abandoned. When the WBC counts were <1000/mm^2^ or the platelet counts were <25,000/mm^2^ after chemotherapy, the doses of 5-FU and CDDP were reduced by 25% at the next administration. The doses of CDDP could be reduced by 25% when serum creatinine levels temporarily exceeded 1.5 mg/dL, the dose of the next CDDP administration alone was decreased by 25%.

Using a 6-MV photon beam by linear accelerator, RT was performed with a daily fraction of 1.8 Gy from day 10 to 37 and 2 Gy from day 49 to 71. The initial radiation field covered between the nasopharynx and middle cervical region, using bilateral opposing portals and between lower cervical and supraclavicular regions using the anterior single field irradiation at a dose of 36 Gy/20 fraction. Then, a shrinking field of 34 Gy/17 fraction was boosted to the nasopharynx using the dynamic conformal rotational technique or bilateral opposing portals. In the cervical lymph nodes in which metastases were detected during the initial examination, 34 Gy of 6-MV photon beam or electron beam in 17 fractions was additionally irradiated (Field B). The dose of irradiation to the spinal cord, optic chiasm, and brain was limited to less than 40 Gy, 48 Gy, and 56 Gy, respectively.

Neck dissection was allowed to be performed on patients with progressive nodal disease or disease persistence.

### Patient assessments

According to the National Cancer Institute-Common Toxicity Criteria ver. 3.0, the toxicity of this ALCRT was evaluated. The antitumor effects of were evaluated 1 month after the completion of this treatment, according to the Response Evaluation Criteria in Solid Tumors (RECIST).

Patients were assessed every 2 months during the first year, every 3 months for the subsequent 2 years, and every 4–6 months thereafter. Locoregional recurrences were assessed by physical examination, a fiberscopic examination, biopsy, and MRI and/or CT. Distant metastases were assessed by chest X-P, liver ultrasonography or CT, and bone scan.

Continuous and categorical variables are presented as medians with ranges and as frequencies with percentages, respectively. OS, progression-free survival (PFS), locoregional recurrence-free (LRF), and distant metastasis-free (DMF) curves were estimated using the Kaplan–Meier method. In univariate analysis, potential factors associated with each of OS, PFS, LRF, and DMF were explored using the log-rank test. In multivariate analysis, the factors that were found to have values of *P* < 0.1 in the log-rank analysis were included in the Cox proportional-hazards regression model. The results are summarized as hazard ratios, 95% confidence intervals, and *P*-values, based on the Cox regression. All P values were two-sided, and *P* < 0.05 was considered statistically significant. Statistical analyses were performed using R (version 3.1.1, Vienna, Austria).

## Results

Ninety eligible patients with NPC were enrolled in this study between December 2003 and March 2006, and 88 patients were finally enrolled, excluding two patients with heart disease and patient refusal. Furthermore, distant metastasis was diagnosed in one patient with the imaging performed during the first chemotherapy, and this patient underwent treatment, according to the protocol. We then evaluated the adverse effects in these 88 patients and the tumor response and survival periods in 87 patients, excluding the distant metastasis case. The patient characteristics were shown in Table1.

**Table 1:** Patient characteristics

Characteristic	Number of patients (%)
Performance status	
0	74 (84.1)
1	12 (13.6)
2	2 (2.3)
Gender	
Male	67 (76.1)
Female	21 (23.9)
Stage	
II B	22 (25.0)
T1N1M0: 11, T2aN1M0: 5,	
T2bN1M0: 6	
III	36 (40.9)
T1N2M0: 13, T2bN2M0: 9,	
T3N0M0: 3 T3N1M0: 2, T3N2M0: 9	
IV A	16 (18.2)
T4N0M0: 3, T4N1M0: 3, T4N2M0: 10	
IV B	13 (14.8)
T1N3M0: 6, T2aN3M0: 1, T2bN3M0:	
1 T3N3M0: 2, T4N3M0: 3	
IV C	1 (1.1)
T3N2M1: 1	
Histology	
WHO type I	18 (20.5)
WHO type II	32 (36.4)
WHO type III	38 (43.2)

Thirteen patients (14.8%) discontinued treatment for the following reasons: unable to continue treatment due to deterioration in the general physical condition (three patients), patient refusal (five patients), responsible physician’s judgment (four patients), and unknown (one patient). Patients were considered to have completed the course of treatment if they were administered at least 70% of both the medication and radiological doses. As a result, a total of 80 patients (91%) completed the radiation therapy, and a total of 67 patients (76.1%) completed the course of treatment. The total tumor dose ranged from 36 to 76 Gy (median: 70 Gy, mean: 69.38 Gy) in the nasopharynx and from 36 to 76 Gy (median: 66 Gy, mean: 64.28 Gy) in the metastatic cervical lymph nodes. The total 5-FU dose ranged from 3380 to 12,669 mg/m^2^ (median: 12,000 mg/m^2^, mean: 10,800 mg/m^2^), and the total CDDP dose ranged from 100 to 306 mg/m^2^ (median: 300 mg/m^2^, mean: 267.5 mg/m^2^). Furthermore, the total duration of RT ranged from 35 to 127 days (median: 66 days), and the overall treatment time (OTT) of ALCRT ranged from 7 to 114 days (median: 87 days). The median follow-up duration for all 88 patients was 67 months (range: 3–91 months) by March 2012.

### Toxicity

The acute toxicities were shown in Table2. The major acute adverse effects of >Grade 3 were as follows: anorexia in 42 patients (47.7%), leukocytopenia in 40 patients (45.5%), and mucositis in 28 patients (31.8%).

**Table 2:** Acute adverse effects

	Toxicity grade (number of patients), *n* = 88				
Toxicity	0	1	2	3	4
Hematologic					
White blood cell	3	9	36	38	2
Neutrophil	22	9	37	20	0
Platelet	31	35	18	5	2
Hemoglobin	7	32	32	12	5
GOT	52	30	5	1	0
GPT	33	44	10	1	0
Creatinine	52	24	12	0	0
Nonhematologic					
Allergic reaction	84	1	3	0	0
Hearing	77	0	9	2	0
Fever	81	0	3	4	0
Infection	4	15	35	28	5
Loss of hair	55	32	1	0	0
Anorexia	7	18	20	42	0
Diarrhea	62	9	15	2	0
Dry mouth	25	27	23	13	0
Mucositis	12	14	34	28	0
Nausea	4	22	39	23	0
Vomiting	33	16	29	10	0
Neurological disorder	86	1	0	0	0

The main late toxicities were shown in Table3. The late adverse effects of >Grade 2 were as follows: dry mouth in 32 patients (36.3%), mucosal damage in 9 patients (10.2%), cervical cellulitis in 1 patient (1.1%), infection of the middle ear in 1 patient, and hearing loss in 1 patient.

**Table 3:** Late adverse effects

	Toxicity grade (number of patients), *n *= 88				
Toxicity	0	1	2	3	4
Temporomandibular joint	87	1	0	0	0
Mucosa	55	14	8	1	0
Dry mouth	39	17	30	2	0
Skin	77	10	0	0	0
Subcutaneous tissue	86	1	1	0	0
Infection					
Cervical cellulitis	87	0	0	1	0
Otitis media	87	0	1	0	0
Hearing	86	1	1	0	0
Brain necrosis	87	1	0	0	0

### Treatment outcome

A complete response was achieved in 54 patients (62.0%), a partial response in 26 patients (29.9%), a stable disease in 4 patients, an unevaluated response in 1 patient, and an unknown in 2 patients. The initial recurrent sites were the nasopharynx in 12 patients, the regional lymph nodes in 3 patients, and distant metastasis in 12 patients. Table4 shows the initial recurrent sites, according to the histopathological findings.

**Figure 2:**
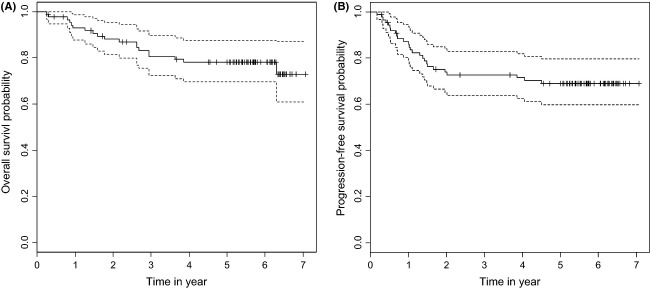
Actuarial survival rates in all 87 patients with nasopharyngeal carcinoma by Kaplan–Meier method. (A) A solid line: overall survival curve. A broken line: 95% confidence interval. (B) A solid line: progression-free survival curve. A broken line: 95% confidence interval.

Until March 2012, 61 patients (70.1%) were alive, and 59 of these 61 remain cancer free. Twelve patients died of local recurrence, 12 patients died of distant metastases, and 2 patients died of unrelated causes without cancer. The OS and PFS curves are shown in Figure2. The 5 year OS and PFS rates were estimated to be 78.04% (95% CI, 69.1–87.0%) and 68.74% (95% CI, 58.8–78.7%), respectively).

**Table 4:** Initial recurrent sites according to WHO histology

	Initial recurrent sites		
WHO histology	Nasopharynx (*n* = 12)	Lymph node (*n* = 3)	Distant metastasis (*n* = 12)
Type I *n* = 18	4	2	0
Type II *n* = 32	4	1	6
Type III *n* = 37	4^1^	0	6

1 One case was maxillary sinus which was out of RT field.

### Factors involved in the overall survival and progression-free survival time (Tables6)

**Table 5:** Results of the univariate analysis of prognostic factors on overall and progression-free survival

		Overall survival			Progression-free survival		
		Univariate analysis					
Factor	Level (*n*)	HR^1^	95% CI	*P*-value	HR^1^	95% CI	*P*-value
Age	<50 (36)	1	0.732–3.476	0.5097	1	0.582–2.826	0.5368
	≥50 (60)	1.367			1.282		
Gender	Male (66)	1	0.732–3.476	0.0937	1	0.049–0.873	0.0176
	Female (21)	0.306			0.206		
TNM primary	1 or 2 (52)	1	0.463–2.866	0.7609	1	0.913–4.272	0.0781
	3 or 4 (35)	1.159			1.975		
TNM LN	0 or 1 (33)	1	1.688–94.785	0.0014	1	2.124–38.12	0.0003
	2 or 3 (54)	12.647			8.998		
Stage	II or III (58)	1	0.6103–3.779	0.3656	1	0.878–4.110	0.0975
	IV (29)	1.518			1.900		
WHO pathology	I (18)	1	0.301–2.745	0.8669	1	0.305–1.895	0.556
	II or III (69)	0.910			0.761		
PS	0 (73)	1	0.125–2.370	0.4109	1	0.470–3.3090	0.6561
	1 or 2 (14)	0.545			1.248		
RT dose primary	<70 (5)	1	0.1518–8.583	0.8989	1	0.172–3.84	0.6654
	≥70 (81)	1.1140			0.728		
RT dose lymph node	<70 (21)	1	0.591–11.072	0.1927	1	0.504–3.545	0.5585
	≥70 (65)	2.558			1.337		
Total dose of 5-FU	<12,000 (37)	1	0.140–0.903	0.0230	1	0.162–0.789	0.0078
	≥12,000 (50)	0.355			0.357		
Total dose of CDDP	<300 (38)	1	0.232–1.411	0.4560	1	0.205–0.974	0.0375
	≥300 (49)	0.573			0.447		
OTT^2^	<87 (38)	1	0.191–1.273	0.1361	1	0.261–1.2691	0.1656
	≥87 (47)	0.494			0.576		

1 Hazard ratio.

2 Overall treatment time.

**Table 6:** Results of the multivariate analysis of prognostic factors on overall and progression-free survival

		Overall survival			Progression-free survival		
		Multivariate analysis					
Factor	Level (*n*)	HR^1^	95% CI	*P*-value	HR^1^	95% CI	*P*-value
Gender	Male (66)	1	0.074–1.404	0.1316	1	0.049–0.910	0.0369
	Female (21)	0.322			0.211		
TNM primary	1 or 2 (52)				1	0.781–3.985	0.1741
	3 or 4 (35)				1.765		
TNM LN	0 or 1 (33)	1	0.097–1.353	0.0243	1	1.453–27.946	0.0141
	2 or 3 (54)	10.267			6.372		
Stage	IIB or III (58)				1	0.517–2.774	0.6734
	IV (29)				1.198		
Total dose of 5-FU	<12,000 (37)	1	0.155–1.012	0.0531	1	0.162–1.015	0.0539
	≥12,000 (50)	0.396			0.406		
Total dose of CDDP	<300 (38)				1	0.358–2.181	0.7889
	≥300 (49)				0.884		

1 Hazard ratio.

In the univariate analysis, N classification and total 5-FU dose had significant impacts on OS. In the multivariate analysis, N classification had a significant impact on OS.

In the univariate analysis, gender, N classification, total 5-FU dose, and total CDDP dose had significant impacts on PFS. In the multivariate analysis, gender and N classification had significant impacts on PFS.

### Factors involved in the locoregional recurrence-free and distant metastasis-free rates (Tables8)

**Table 7:** Results of the univariate analysis of prognostic factors on locoregional and distant metastasis-free rates

		Locoregional-free rate			Distant metastasis-free rate		
		Univariate analysis					
Factor	Level (*n*)	HR^1^	95% CI	*P*-value	HR^1^	95% CI	*P*-value
Age	<50 (36)	1	0.4715–5.200	0.4604	1	0.330–3.278	0.9462
	≥50 (50)	1.566			1.040		
Gender	Male (66)	1	0, infty	0.0303	1	0.116–2.417	0.4037
	Female (21)	Near 0			0.529		
TNM primary	1 or 2 (52)	1	0.956–10.548	0.0464	1	0.492–4.735	0.4602
	3 or 4 (35)	3.175			1.527		
TNM LN	0 or 1 (33)	1	0.761–15.925	0.0866	1	0, infty	0.0032
	2 or 3 (54)	3.482			Infty		
Stage	IIB or III (58)	1	0.318–3.518	0.9262	1	1.390–15.379	0.0060
	IV (29)	1.058			4.624		
WHO pathology	I (18)	1	0.099–0.986	0.0359	1	0, infty	0.0850
	II or III(69)	0.312			infty		
PS	0 (73)	1	0.476–6.503	0.3902	1	0.252–5.250	0.8572
	1 or 2 (14)	1.760			1.149		
RT dose primary	<70 (5)	1	0.064–1.340	0.0924	1	0, infty	0.4078
	≥70 (81)	0.293			infty		
RT dose LN	<70 (21)	1	0.254–3.471	0.9255	1	0.356–7.407	0.5279
	≥70 (65)	0.940			1.622		
Total dose of 5-FU	<12,000 (37)	1	0.135–1.349	0.1354	1	0.143–1.429	0.1657
	≥12,000 (50)	0.427			0.453		
Total dose of CDDP	<300 (38)	1	0.210–2.024	0.4560	1	0.096–1.060	0.0490
≥300 (49)	0.652				0.319		
OTT^2^	<87 (38)	1	0.126–1.474	0.1673	1	0.238–2.291	0.5986
	≥87 (47)	0.431			0.739		

1 Hazard ratio.

2 Overall treatment time.

**Table 8:** Results of the multivariate analysis of prognostic factors on locoregional and distant metastasis-free rates

		Locoregional-free rate			Distant metastasis-free rate		
		Multivariate analysis					
Factor	Level	HR^1^	95% CI	*P*-value	HR^1^	95% CI	*P*-value
TNM primary	1 or 2 (52)	1	0.897–10.370	0.0740			
	3 or 4 (35)	3.051					
TNM LN	0 or 1 (33)	1	0.531–11.913	0.2454			2
	2 or 3 (54)	2.514					
Stage	IIB or III (58)				1	1.549–17.347	0.0076
	IV (29)				5.184		
WHO pathology	I (18)	1	0.074–0.901	0.0336			3
	II or III(69)	0.258					
RT dose primary	<70 (21)	1	0.035–0.972	0.0463			
	≥70 (65)	0.183					
Total dose of 5-FU	<12,000 (37)	1	0.155–1.012	0.0531			
	≥12,000 (50)	0.396					
Total dose of CDDP	<300 (38)				1	0.082–0.925	0.0369
	≥300 (49)				0.2762		

Gender was not incorporated into the multivariate analysis because no events were observed in female.

1 Hazard ratio.

2 N classification was not incorporated into the multivariate analysis because no events were observed in N0 or N1 patients.

3 WHO histology classification was not incorporated into the multivariate analysis because no events were observed in WHO type I patients.

**Figure 3:**
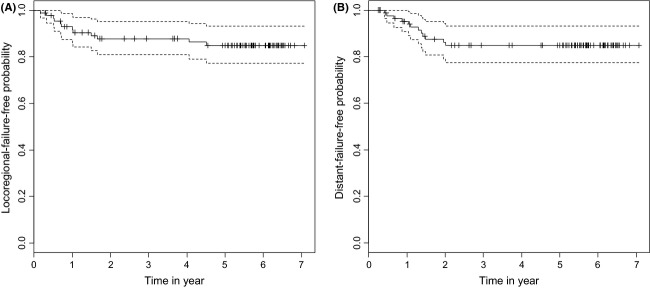
(A) Actuarial locoregional recurrence-free (LRF) rates in all 87 patients with nasopharyngeal carcinoma by Kaplan–Meier method. A solid line: LRF curve. A broken line: 95% confidence interval. (B) Actuarial distant metastasis-free (DMF) rates in all 87 patients with nasopharyngeal carcinoma by Kaplan–Meier method. A solid line: DMF curve. A broken line: 95% confidence interval.

The LRF and DMF curves are shown in Figure3. In the univariate analysis, gender, T classification, and WHO histological classification had significant impacts on the LRF rate. In the multivariate analysis, the WHO histological classification and radiation dose in the nasopharynx had significant impacts on LRF rate.

In the univariate analysis, N classification, stage, and total CDDP dose had significant impacts on the DMF rate. In the multivariate analysis, stage, total CDDP dose, N classification, and WHO histological classification had significant impacts on the DMF rate.

## Discussion

IGS 0099 was considered a landmark for NPC treatment 7. However, 3 courses of AC were administered after concurrent chemoradiotherapy (CCRT) in 55% of the patients. The objective of chemotherapy in NPC treatment is not only the improvement of local control but also the control of micrometastasis. In particular, the main role of AC is to control the latter, that is, distant metastasis. An analysis that combined data from NPC 9901 and NPC 9902, large-scale clinical trials involving the same treatment method as that outlined in IGS 0099, has indicated that AC was a factor related to the control of distant metastasis and that a particular correlation was noted with the 5-FU dose. When compared with 260 patients who received 0–1 cycle of adjuvant cycles, significant improvement was achieved in 167 patients with 3–4 cycles (*P* = 0.019) 10. A meta-analysis also found that the involvement of CCRT in the control of distant metastasis was marginal (*P* = 0.04), but AC was a factor that exhibited a significant involvement (*P* = 0.009) 11. In accordance, AC has been shown to be involved in the decrease of distant metastasis, but the results of a randomized controlled trial (RCT) comparing the treatment outcomes between a CCRT + AC group and a CCRT group and the results of a meta-analysis have not demonstrated any improvements in survival rates by the addition of AC 12,13.

It has been proved that acute toxicity caused by CCRT reduces tolerance to AC, and neoadjuvant chemotherapy (NAC) + CCRT may be one future direction for treatment. Boscolo et al. reported the favorable long-term retrospective outcome after NAC+CCRT for locally advanced NPC 14. In 2009, Hui et al. divided subjects into a group that underwent NAC involving 2 courses of CDDP (75 mg/m^2^) and docetaxel (75 mg/m^2^) every 3 weeks before undergoing CDDP (40 mg/m^2^) weekly as CCRT and a group that underwent CCRT with the same method. They compared these groups in a randomized phase II study and found that the 3-year OS rates were 94.1% and 67.7% for each group, respectively, and the 3-year PFS rates were 88.2% and 59.5%, respectively, indicating favorable outcomes for the NAC + CCRT group 15. However, in 2013, Liang et al. conducted 11 RCTs comparing groups that underwent CCRT after NAC with those that underwent CCRT (with or without AC) in 1096 patients in a meta-analysis and found that PFS was improved, but no differences were observed for OS, locoregional failure-, and DMF survival 16. Taxane (docetaxel or paclitaxel) has been confirmed in a meta-analysis as an effective medication for NAC in patients with advanced carcinomas of the head and neck 17. In 7 of the 11 above-mentioned RCTs, taxane was used. These findings suggest that NAC increases the repopulation of the tumor and a buildup of chemoresistant tumor cells, resulting in decreased therapeutic results in CCRT 18.

At present, it is clear that CCRT improves the survival rates more than RT alone 12,19. The addition of AC after CCRT does not lead to a greater improvement in the survival rates compared with CCRT without AC 12,13. It is unclear whether the addition of NAC to CCRT improves survival rates compared with CCRT alone.

Compared with other carcinomas of the head and neck, NPC has high potential of distant metastasis 2. Insufficient dose chemotherapy has not shown a benefit of control of distant metastasis compared with RT alone, which was suggested in Chan’s study (RT concurrently with cisplatin at a dose of 40 mg/m^2^ weekly, *P* = 0.15) 21. It is characteristic in NPC that a wider irradiation field is required during RT due to its progression pattern, and mucositis is a marked problem even when RT alone is performed. Considering these characteristics of NPC, ALCRT has two advantages compared with CCRT; a sufficient amount of antitumor agents can be administered to control distant metastasis, and it has the potential benefit in reducing acute toxicities 7,9.

In Japan, the proportion of patients with WHO type I histopathology is approximately 20%, which is similar to that in North America and much higher than the rates in studies conducted in endemic regions 23. It has proved to be less sensitive to RT and a significantly worse prognostic factor of OS and PFS 21,22.

The ALCRT that we administered in the present study led to a 76% treatment completion rate, 78% 5-year OS, and 69% 5-year PFS. Compared with IGS 0099, the ALCRT was advantageous in these followings; a decreased total dose of CDDP (540 mg/m^2^ vs. 300 mg/m^2^), a shorter treatment period (130 days vs. 87 days), a higher treatment completion rate (53% vs. 76%), and a higher survival rate (78% 3y-OS in IGS 0099 vs. 78% 5y-OS in this study, 69% 3y-PFS in IGS 0099 vs. 69% 5y-PFS in this study). Considering that the percentage of patients classified as WHO type I, indicating a poor outcome, was 21%, the above-mentioned treatment outcomes appear to be extremely favorable.

The present study found that WHO type I histopathology recurred in 6 of the 18 patients, and that recurrence occurred from the irradiation field in all the patients. Therefore, as shown in Table4, in order to improve the local control rate, a higher dose RT in the form of intensity-modulated RT (IMRT) which is helpful for improving clinical results is necessary, especially for patients who exhibit WHO type I histopathology 23. Recurrence occurred in 20 of the 69 patients who exhibited WHO type II and III histopathology. Twelve of these patients exhibited distant metastasis, suggesting the necessity of a stronger anti-cancer drug treatment or the combined use of molecular targeted therapy.

## Conclusions

This method of ALCRT yielded higher or at least similar survival rates and lower toxicities compared with CCRT. The future direction of ALCRT appears likely to involve the introduction of IMRT, and concerning anti-cancer drugs, the addition of taxane to conventional FP. We believe that ALCRT will be used in a controlled trial to compare therapeutic results with those of CCRT (with or without NAC).
